# DNMT3B Expression Might Contribute to Abnormal Methylation of RASSF1A in Lager Colorectal Adenomatous Polyps

**DOI:** 10.1155/2020/1798729

**Published:** 2020-10-01

**Authors:** Xianmei Meng, Na Liu, Yanbin Jia, Kerui Gong, Jingjie Zhang, Wei Zhang, Guo Shao, Tong Dang

**Affiliations:** ^1^Department of Gastroenterology, The Second Affiliated Hospital of Baotou Medical College, Baotou, 014030 Inner Mongolia Autonomous Region, China; ^2^Institute of Digestive Diseases of Inner Mongolia Autonomous Region, Baotou, 014030 Inner Mongolia Autonomous Region, China; ^3^Inner Mongolia Key laboratory of Hypoxic Translational Medicine, Baotou Medical College, Baotou, 104060 Inner Mongolia Autonomous Region, China; ^4^Department of Oral and Maxillofacial Surgery, University of California San Francisco, San Francisco, 94122 CA, USA

## Abstract

**Background:**

It is pretty well known that DNA methyltransferases (DNMTs) are actively involved in abnormal cell growth. The goal of the current study is to explore the correlation between DNMT expression and colorectal adenomatous polyps (CAPs).

**Method:**

Twenty pairs of CAP samples with a diameter ≥ 10 mm and corresponding normal colorectal mucosa (NCM) tissues from patients were used in the present study. The expression levels and activity of DNA methyltransferases (DNMTs) were measured in the CAP tissues. The global methylation and the promoter methylation level of 3 kinds of tumour suppressor gene were detected.

**Results:**

mRNA and protein levels of *DNMT3B* were found to be elevated in the CAP tissues compared with the control tissue. Additionally, the methylation of long interspersed nuclear elements-1 (*LINE-1/L1*) was decreased in the CAP tissue. Furthermore, methylation of the promoter of a tumour suppressor gene Ras association domain family 1A (*RASSF1A*) was increased in the CAP tissues, while the mRNA levels of *RASSF1A* were decreased.

**Conclusions:**

These results suggest that the overexpression of *DNMT3B* may contribute to a role in the genesis of CAPs through the hypomethylation of chromosomes in the whole cell and promoter hypermethylation of *RASSF1A*.

## 1. Introduction

Colorectal cancer (CRC) ranks as the fifth and third most commonly diagnosed cancer in the Chinese and American populations [1, 2]. Around the world, CRC is the third leading cause of cancer-related death. Colorectal adenomatous polyps (CAPs) are regarded as CRC precursors and known to be precancerous lesions. Stryker et al. revealed that a very small number of polyps that were initially 2–5 mm in diameter would eventually become invasive carcinomas. They suggested that the diminutive size of most of these polyps might be hyperplastic rather than adenomatous and, thus, not at risk for malignant change. While, in comparison, some colonic polyps ≥ 10 mm in diameter were eventually shown to harbour invasive carcinoma, and the risk of carcinoma approached 25% at 20 years [3]. It has been widely accepted that there are multiple transformations in the colorectal adenoma–carcinoma sequence. Both genetic and epigenetic mechanisms play roles in the stepwise progression from normal to dysplastic epithelium and to carcinoma [4–6].

The silence of tumour suppressor genes or tumour-related genes is one important mechanism caused by epigenetics, which may act as an alternative to genetic mutations, in molecular carcinogenesis [7]. Abnormal epigenetic alterations of DNA methylation should be considered to be a hallmark of carcinomas. DNA methylation belongs to postsynthetic modifications, which take place at the carbon-5 position of cytosine nucleotides in CpG dinucleotides and are regulated by DNA methyltransferase (DNMT). Increases in DNMT expression have been found in polyps and may be regarded as a remarkable accrual of genetic instability affairs, accompanied with early events in cell transformation [8]. It is well known that three DNMTs, including DNMT1, DNMT3A, and DNMT3B, are responsible for directing mammalian genomic methylation patterns. While DNMT1 works primarily as a maintenance enzyme, DNMT3A and DNMT3B work as *de novo* enzymes [9]. Public dataset (https://www.proteinatlas.org) showed that high expression of DNMT1, DNMT3A, and DNMT3B was 46%, 74%, and 80% in CRC, respectively. And DNMT levels correlated significantly with the reduced survival probability. However, it still remains obscure about which DNMTs contribute to the risk of malignant change.

It has been accepted that some colonic polyps ≥ 10 mm have more risk of progression into invasive carcinoma [3]. To clarify the role of DNMTs in the stepwise progression from normal to dysplastic epithelium and to CRC, samples of CAPs ≥ 10 mm were collected and the expression of DNMTs was measured. Carcinogenesis was associated with changes in two distinct opposing DNA methylation affairs: hypermethylation in antioncogene and hypomethylation in global methylation. Long interspersed nucleotide elements (*LINE*) are 6–8 kb long, have GC-poor sequences, and make up 15% of the human genome. Alu-repetitive elements are shorter, about 300 bp in length, are GC-rich, and make up 10% of the human genome [10]. The methylation level of *LINE-1* or *Alu* can be regarded as the global genomic methylation level. Simultaneously, the methylation levels of *LINE-1* or *Alu* in these samples were detected. Furthermore, the methylation levels of promoters for these three tumour suppressors were estimated. In this study, we revealed that *DNMT3B* was increased; *LINE-1* methylation levels were decreased, while the promoter of *RASSF1A* was hypermethylated, and its expression was decreased. These epigenetic affairs may be responsible for the formation of bigger colorectal adenomatous polyps, which may be a key step in the adenoma–carcinoma sequence.

## 2. Materials and Methods

### 2.1. CAP Tissue Sample Collection

Twenty pairs of tissue samples were acquired from CAP patients who had undergone endoscopic resection of polyps between 2017 and 2018 at the Second Affiliated Hospital of Baotou Medical College (Table 1). Consent forms were signed by all participating patients, and this study was approved by the College Ethics Committee.

### 2.2. Real-Time PCR

Total RNA was prepared using a RNeasy mini kit (Qiagen, Valencia, CA, USA) from CAP tissue and normal colorectal mucosa (NCM) tissue. cDNA synthesis was performed using a Superscript III FIRST Strand synthesis kit (Invitrogen, Carlsbad, CA, USA). The following gene-specific PCR primers Table 2 were used for real-time PCR:

All PCR reactions were performed on an ABI-7900 real-time PCR machine with the protocol as previously described [11]: initial denaturation at 95°C for 10 min, 40 cycles at 95°C for 30 sec, 40 cycles at 60°C for 60 sec, and a final extension at 60°C for 2 min in a 50 *μ*l reaction mixture containing 2 *μ*l of each cDNA, 0.2 *μ*M of each primer, and 25 *μ*l 2 X real-time master mix. Real-time analyses were performed in triplicate for each sample-primer set as the CT value. The relative mRNA expression levels were calculated using the DD value (*β*-actin as control) [12]. In order to further confirm the relative mRNA levels of target genes, the fragments of target genes were cloned into the TA vector as standard, and plasmid DNA (0.1 *μ*g) was cut with 10 units of EcoR I (Takara, Dalian, China). The absolute values of target genes mRNA level were measured as Whelan et al. described [13].

### 2.3. Immunoblotting

Protein from CAP tissues and NCM tissues was prepared with a RIPA buffer (Beyotime Institute of Biotechnology, Jiangsu, China), and the protein concentrations were determined by the BCA method (Beyotime Institute of Biotechnology, Jiangsu, China). A total of 15 *μ*g of protein was separated on 12% gel using SDS-PAGE, and then gel proteins were electrically transferred onto a nitrocellulose membrane (Roche, Germany), which was blocked with a blocking buffer containing 10% skimmed milk. Primary antibodies against DNMTs (Novus Biologicals, Littleton, CO, USA) and *β*-actin (Sigma, St. Louis, Mo. USA) were used to bind the target protein. Secondary antibody binding was carried out following the manufacturer's recommendations. Protein signals on membranes were captured and analysed by Tanon 4600 (Biotanon, Shanghai, China).

### 2.4. DNA Methyltransferase Activity Assay

Nuclear proteins from both CAP and NCM tissues were purified using the commercial Kit (Epigentek, Brooklyn, NY, USA). The total DNA methyltransferase activities for DNMT, DNMT1, and DNMT3A were measured following the manufacturer's instructions of the DNA methyltransferase (DNMT) activity assay kit (Epigentek, Brooklyn, NY, USA). DNA methyltransferase activity analysis was conducted on three different samples and performed in triplicate for each sample.

### 2.5. Global DNA Methylation of CAP Tissues

It is well known that methylation of repeated DNA elements (REs), such as *Alu* and *LINE-1* elements, can present the genetic global DNA methylation level. A DNA extraction kit (Qiagen Inc., Valencia, CA, USA) was used to isolate DNA from the CAP and NCM tissues. The methylation levels of *Alu* and *LINE-1* were measured by combined bisulfite restriction analysis (COBRA), after the DNA was treated by bisulfite (ZYMO Research, Irvine, CA). The primers of *Alu* and *LINE-1*, PCR cycling conditions, and analysis of COBRA product were performed as previously described [11].

### 2.6. *hMLH1*, *P16*, and *RASSF1A* Methylation-Specific PCR (MS-PCR)

*hMLH1*, *P16*, and *RASSF1A* methylation was determined by sodium bisulfite treatment of DNA as described above, followed by MS-PCR. The MS-PCR primers Table 3 and the experimental conditions that were used were identical to those reported [14–16].

Nested PCR was performed, and the results of the methylation levels were analysed as previously described [11]. PCR product (5 ul) was detected by 4% agarose gel electrophoresis with ethidium bromide (EtBr) using ultraviolet (UV) light in a transilluminator.

### 2.7. Quantification and Statistical Analysis

The resulting data of immunoblotting bands and PCR experiments were collected and analysed as previously described [11]. All data are expressed as mean ± standard deviation (SD). Analysis of variance (ANOVA) or Tukey's HSD test was used for statistical analysis. A *P* value < 0.05 was considered to be statistically significant.

## 3. Results

### 3.1. *DNMT3B* Expression Levels Increased in CAP Tissues

**Figure 1:**
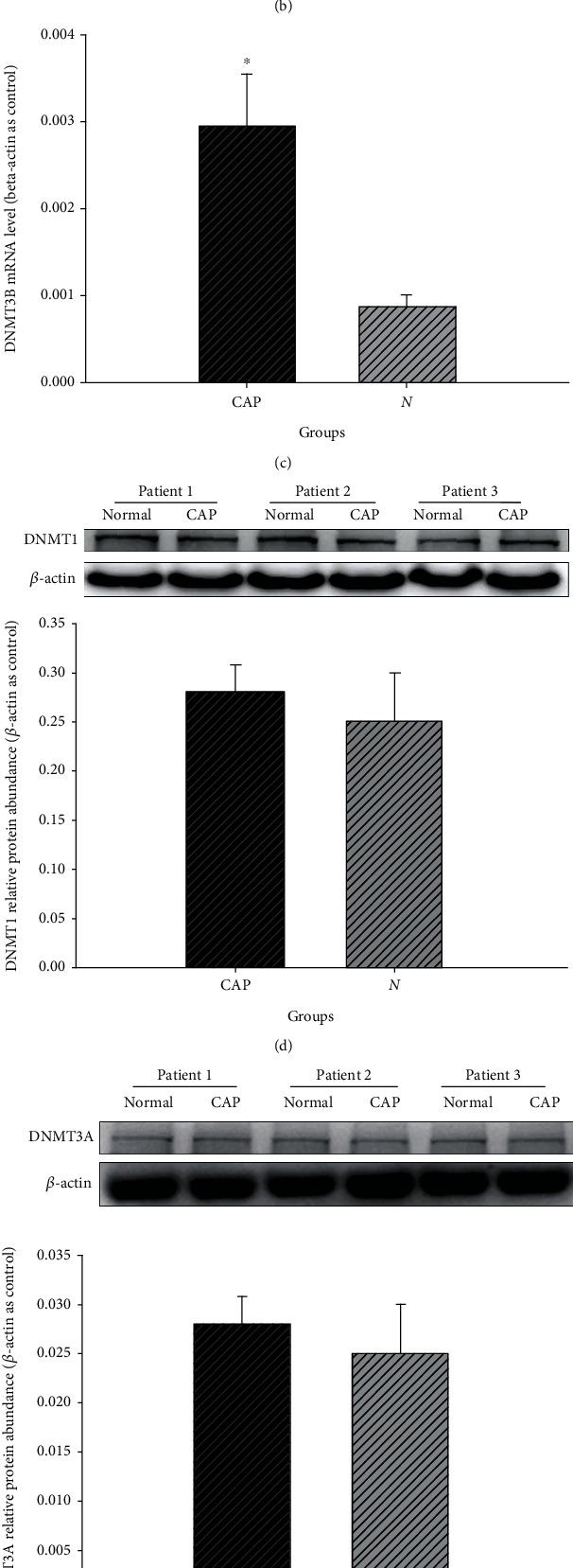
The analysis of DNMT expression levels in CAP and NCM tissues.The relative expression for the mRNA of DNMT1 (a), DNMT3A (b), and DNMT3B (c). The relative protein level for DNMT1 (d), DNMT3A (e), and DNMT3B (f) determined by western blotting protein. (^∗^*P* < 0.05).

To measure the expression levels of DNMTs (*DNMT1*, *DNMT3A*, and *DNMT3B*) in CAP tissues and NCM tissues, we analysed 20 pairs of tissue samples, which included CAP tissue and NCM tissue (obtained sample 10 cm from CAPs [17]), using real-time PCR. Relative expression levels of *DNMT3B* mRNA were significantly higher in the CAP tissues than those in the NCM tissue (*P* < 0.05). While, in comparison, no statistically significant difference was observed in relative mRNA levels of *DNMT1* and *DNMT3A* between the CAP and NCM tissues (Figures 1(a)–1(c)) (*P* > 0.05). At the same time, absolute mRNA expression levels of *DNMT1*, *DNMT3A*, and *DNMT3B* were similar to that of relative mRNA expression levels (Supplementary Figure 1).

DNMT3B protein levels were measured by immunoblotting. The expression of DNMT3B can be detected in both CAP and NCM tissues (Figure 1(f)). Compared with NCM tissues, DNMT3B protein was found to be increased (CAP/NCM tissues > 1) in 60% (12/20) of the CAP tissues. DNMT3B protein was unchanged or slightly decreased in 40% (8/20) of the other CAP tissues. There was a significant difference in the expression of DNMT3B (*P* < 0.05), with no significant difference in DNMT1 and DNMT3A between CAP and NCM tissues (Figures 1(d) and 1(e)). Therefore, consistent with the result from real-time PCR, our data found a higher expression of DNMT3B in CAP tissue than NCM tissue (Figure 1(f)).

### 3.2. CAP Tissues Exhibit More DNMT3B Activity than NCM Tissue

**Figure 2:**
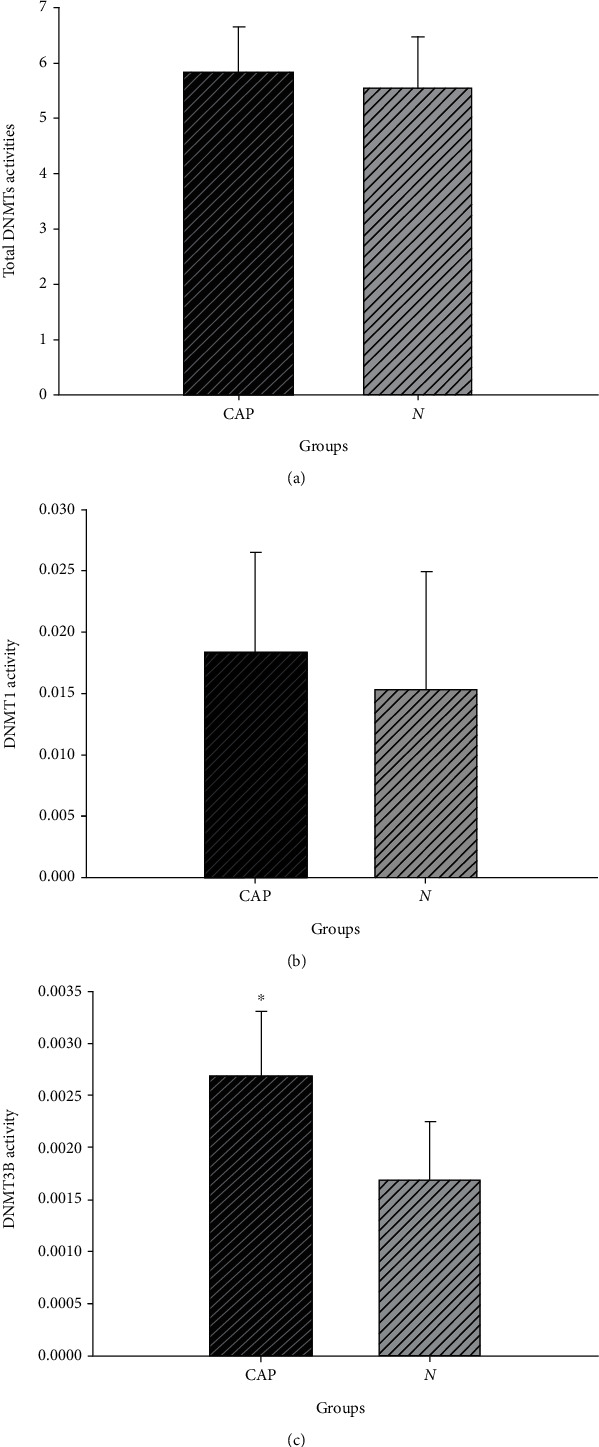
The analysis of DNMTs activity in CAP and NCM tissues. (a–c) showed the total DNMTs, DNMT1, and DNMT3B activities, respectively. (^∗^*P* < 0.05).

The total activity of DNMT, DNMT1, and DNMT3B in CAP issue and NCM tissues was examined by ELISA using a commercial DNA methyltransferase activity assay kit. No statistically significant differences in the total DNMT and DNMT1 activity between the CAP and NCM tissues were found (Figures 2(a) and 2(b)). In contrast, as shown in Figure 2(c), DNMT3B activity in the CAP tissues (0.0028 ± 0.00062) was higher than the NCM tissues (0.0017 ± 0.00056) (*P* < 0.05).

### 3.3. CAP Tissues Exhibit a Greater Genomic Unmethylation Level than Normal Mucosa Tissue

**Figure 3:**
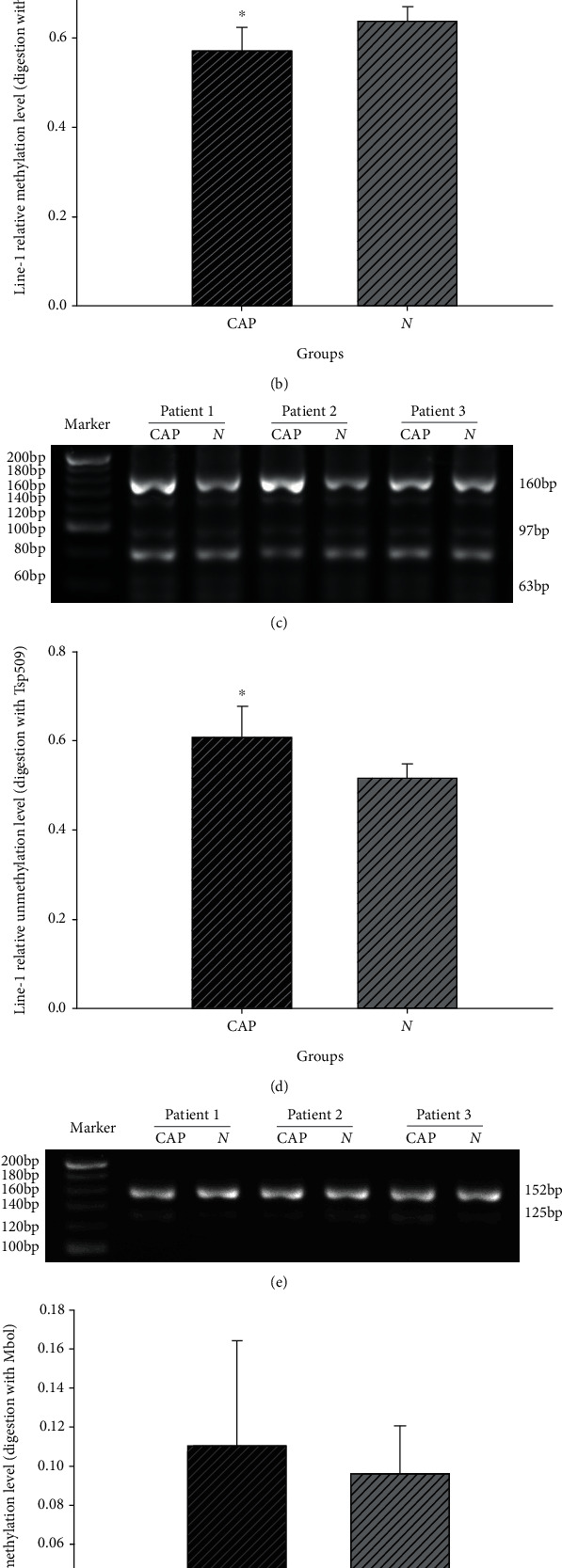
Global methylation of LINE-1 and Alu in CAP and NCM tissues. (a) Representative electrophoresis images of COBRA products (LINE-1 DNA sequence + Taq1 digestion). (b) The statistical analysis showed the rate of DNA methylation in the LINE-1 DNA sequence has a difference between CAP and control tissues(^∗^*P* < 0.05). (c) Representative electrophoresis images of COBRA products (LINE-1 DNA sequence + TSP509I digestion) in agarose gel. (d) The statistical analysis showed the rate of DNA unmethylation in the LINE-1 DNA sequence has a difference between CAP and control tissues(^∗^*P* < 0.05). (e) Representative electrophoresis images of COBRA products (Alu DNA sequence + Mbo1 digestion). (f) The statistical analysis showed the rate of DNA methylation in Alu DNA sequence did not exhibit any difference between CAP and control tissues.

Methylation levels of *Alu* and *LINE-1* elements, which present the methylation of global genomic DNA, were assessed by COBRA. DNA methylation of *Alu* and *LINE-1* elements was calculated as previously described [11]. The *LINE-1* methylation levels in CAP and NCM tissues were 0.569 ± 0.056 and 0.637 ± 0.034 (Figures 3(a) and 3(b)). The *LINE-1* methylation level was higher in the CAP tissues compared to NCM tissues (*P* < 0.05). Finally, the *LINE-1* unmethylation levels in the CAP and NCM tissues were 0.605 ± 0.072 and 0.515 ± 0.033 (Figures 3(c) and 3(d)). The *LINE-1* unmethylation level was lower in the CAP tissues than in the NCM tissues (*P* < 0.05). There was a difference between the CAP and NCM tissues in *LINE-1* elements DNA methylation and DNA unmethylation levels (*P* < 0.05). The *Alu* methylation levels in the CAP and NCM tissues were 0.110 ± 0.054 and 0.096 ± 0.025. There was no difference between these (*P* > 0.05) (Figures 3(e) and 3(f)).

### 3.4. CAP Tissues Exhibit Hypermethylation in *RASSF1A* Promoter Sequences and Lower Expression of *RASSF1A* mRNA

**Figure 4:**
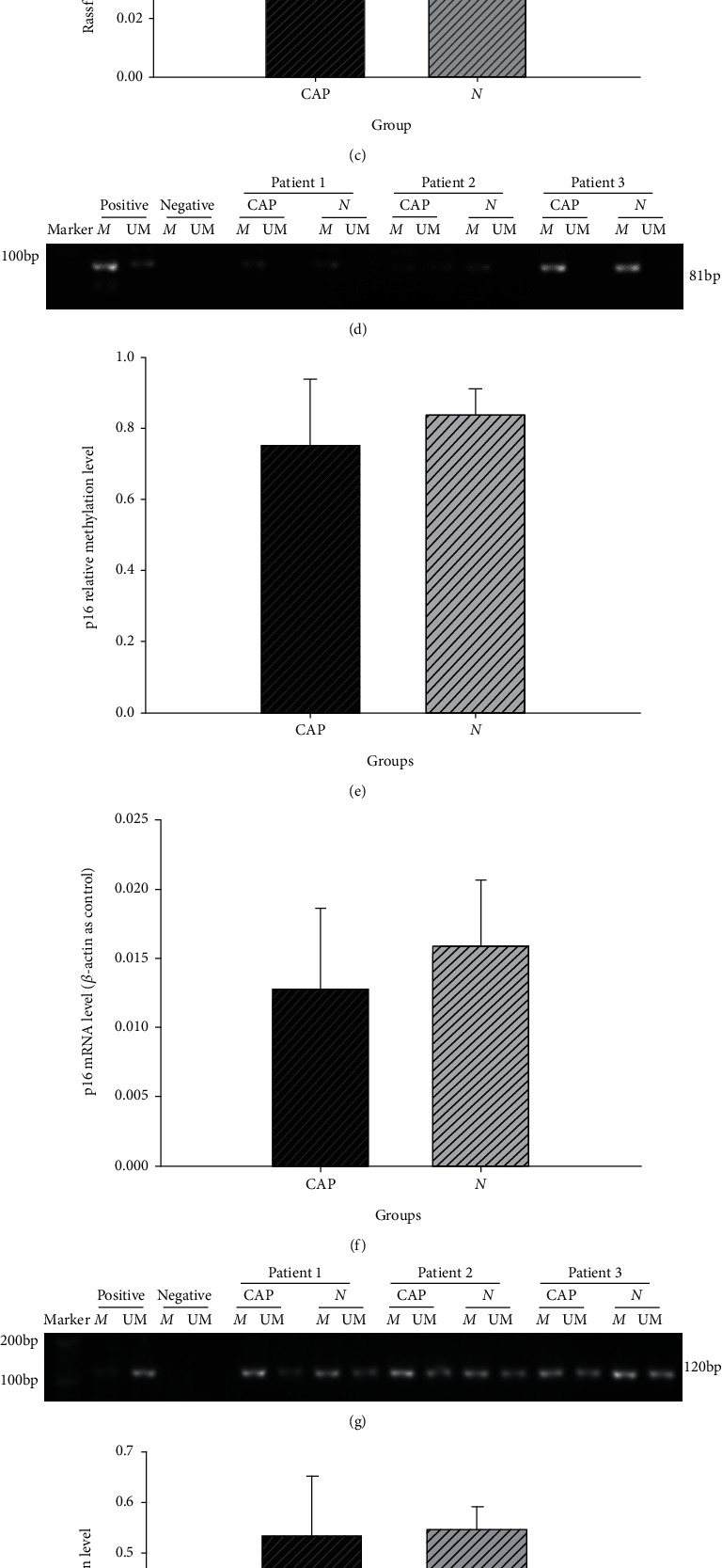
The methylation and expression of *RASSF1A*, *p16*, and *hMLH1* in CAP and NCM tissues. (a) MS-PCR analysis of methylation of *RASSF1A*. (b) Rate of DNA methylation in *RASSF1A* (^∗^*P* < 0.05). (c) Real-time PCR analysis of the ration of *RASSF1A* mRNA to *β*-actin mRNA (^∗^*P* < 0.05). (d) MS-PCR analysis of methylation of *p16*. (e) Rate of DNA methylation in *p16*. (f) Real-time PCR analysis of the ration of *p16* mRNA to *β*-actin mRNA. (g) MS-PCR analysis of methylation of *hMLH1*. (h) Rate of DNA methylation in *hMLH1*. (i) Real-time PCR analysis of the ration of *hMLH1* mRNA to *β*-actin mRNA.

The promoter methylation levels of *RASSF1A*, *P16*, and *hMLH1*were asserted by methylation specific PCR (MS-PCR), and the DNA methylation level was calculated as the OD of methylation products/methylation products + unmethylation products. The promoter methylation levels of *RASSF1A* were increased in the CAP tissues (0.611 ± 0.082) as compared with that in NCM tissues (0.556 ± 0.081). There was a statistically significant difference between these (*P* < 0.05) (Figures 4(a) and 4(b)). No differences in the promoter methylation levels of *P16* (Figures 4(d) and 4(e)) and *hMLH1* (Figures 4(g) and 4(h)) could be discerned between the CAP and NCM tissues (*P* > 0.05).

Real-time PCR was used to detect the mRNA levels of *hMLH1*, *P16*, and *RASSF1A* in the CAP and NCM tissues. *RASSF1A* mRNA expression levels in CAP tissues (0.047 ± 0.007) were found to be nearly two times lower than those of the NCM tissues (0.083 ± 0.016) (*P* < 0.05) (Figure 4(c)). There were no differences in *P16* (Figure 4(f)) and *hMLH1* (Figure 4(i)) mRNA levels in the CAP and NCM tissues (*P* < 0.05).

## 4. Discussion

DNA methylation patterns, catalysed by DNMTs, are characteristically stable in somatic cells and are changeable in cancer cells [11]. Aberrant DNA methylation pattern is one of the most consistent epigenetic changes in human cancers. Generally, cancer cells have features of global DNA hypomethylation. At the same time, hypermethylation was found in some specific gene promoter regions in cancer cells [18, 19]. Interestingly, both the decrease of the global methylation level and increase of some tumour-associated gene promoters were found in CAPs [20, 21]. Therefore, DNMTs may be a critical regulator in the process of multiple alterations in the adenoma–carcinoma sequence. Huang et al. reported that DNMTs were upregulated in para-CRC tissues [17], and it has been well established that total DNMTs were found to be at a 60-fold increase in premalignant CAPs [8]. However, it still remains unclear as to which subtype of DNMT contributes as a pivotal role in the adenoma–carcinoma sequence.

DNA methyltransferase expression and activity in 20 pairs of CAP samples with a diameter ≥ 10 mm was analysed in the current study. mRNA expression of the DNMTs was measured by Q-PCR, with only *DNMT3B* showing significant upregulation levels in the CAP tissues. DNMT3B protein levels were increased in 12/20 of the CAP patients. Similar to our results, using an immunohistochemical method, Ibrahim and his colleagues found that *DNMT3B* expression increased significantly from normal to hyperplastic, from CAP to CRC samples [22]. On the contrary, Eads and his colleagues found DNMTs, including *DNMT3B*, were increased or not in tumours when RNA levels had normalized using different housekeeping gene controls. Their data implied that the upregulation of *DNMT* gene expression did not significantly contribute to the establishment of tumour-specific abnormal DNA methylation patterns in CRC [23]. This discrepancy could be due to mRNA level changes which did not always directly reflect the protein levels. At the same time, *DNMT3B* polymorphism may be responsible for susceptibility to colorectal adenomatous polyps and adenocarcinoma [24, 25]. These results suggest that there is a potential relationship between increased *DNMT3B* expression and tumour transformation from normal cells to conventional adenoma cells.

DNMT expression and global genomic methylation levels of CAPs with a diameter ≥ 10 mm were investigated in this study, and it is well known that some colonic polyps ≥ 10 mm have more risk of becoming an invasive carcinoma [3]. Qasim and colleagues reported that the global genomic methylation level was significantly lower in large size adenomas (≥10 mm) than in small-sized ones [26]. Repetitive DNA elements contain much of the CpG methylation and represent the global genomic methylation level. About 45% of genomic DNA is repetitive DNA elements, such as *ALU* and *LINE-1*, and the methylation levels of repetitive DNA elements have been considered to represent the level of 5-methylcytosine in the genome [27]. The lack of DNA methylation in repetitive DNA elements may be the main cause of global hypomethylation, a feature of most human cancers [27]. Jiang et al. reported that the hypomethylation of *LINE-1* in polyps from colorectal patients was associated with the presence of synchronous CRC [20]. Sarabi and Naghibalhossaini found that there was a positive correlation between the expression of DNMT and the global DNA methylation level in CRC cells [28]. In the current research, the methylation levels of *LINE-1* were decreased in the samples where *DNMT3B* was increased in the CAP tissues (≥10 mm). Therefore, the global genomic methylation level and the expression *DNMT3B* may be involved in the stepwise progression of adenoma–carcinoma. Inhibitors of the DNMTs have been used in a clinical setting in myelodysplastic syndrome [29]. Thus, our findings implied that DNMT inhibitor may be used as a potential epigenetic therapy in larger CAP to interrupt the stepwise progression of adenoma–carcinoma.

Aberrant DNMT expression may increase some antioncogene promoter methylation levels which lead to the silence. In the present study, we focused on the methylation levels of three genes, which have been suggested to play roles in the development of CRC. Human Mut L homologue1 (*hMLH1*), *CDKN2A/p16*, and *RASSF1A* are these genes belonging to DNA repair genes or tumour suppressor. Some reports found that there were aberrant hypermethylation in the promoters of certain tumour suppressor and DNA repair genes, and it silenced the expression of them in CRC and CAPs [30, 31]. It was found that in *hMLH1*, a DNA repair gene, promoter methylation showed a stepwise increase in normal colon mucosa, adenoma, and carcinoma, respectively [31]. The frequency of *CDKN2A/p16* promoter methylation was very rare in normal colorectal tissue, and the hypermethylation of the *CDKN2A/p16* promoter led to the development of invasive carcinomas [32, 33]. *CDKN2A/p16* hypermethylation was found in 38% of CRCs and was not found in CAPs and normal serum [34]. *RASSF1A* is functionally involved in cell cycle control, and its DNA methylation has been associated with CRC development [35, 36]. In the current study, the methylation levels and expression of *hMLH1* and *CDKN2A/P16* did not show any difference between CAP and NCM tissues, while *RASSF1A* expression was decreased and its promoter was hypermethylated. Therefore, the hypermethylation of *RASSF1A* leads to its expression decrease, which may contribute to the development of bigger CAPs. At the same time, Palakurthy et al. proved that the overexpression of *DNMT3B* correlated with the hypermethylation and silencing of *RASSF1A* expression [37]. On the other hand, decreased *DNMT3B* can facilitate the demethylation of the *RASSF1A* promoter and restore its expression [38]. Thus, the overexpression of *DNMT3B* may be responsible for the hypermethylation and silencing of *RASSF1A* in the formation of bigger CAPs.

It is well known that epigenetic and genetic alterations contribute to the process of adenomas to malignant carcinoma. *Kirsten rat sarcoma viral oncogene homolog* (*KRAS*) and *V-raf Murine Sarcoma Viral Oncogene Homolog B1 (BRAF)* were regarded as genetic biomarkers in CRC. *BRAF* is an immediate downstream effector of *KRAS* in the MAPK signaling pathway [39]. It is well known that some potential molecular targets for cancer diagnosis and treatment are in the MAPK signaling pathway, and these targets play important roles in neoplastic gastrointestinal tissues as well [40]. Fujishita et al. found that MEK/ERK signaling plays key roles in intestinal adenoma formation in Apc*Δ*716 mice [41]. Yuen et al. reported that *BRAF* mutations are biologically similar to *RAS* mutations in colorectal cancer because both occur at approximately the same stage of the adenoma-carcinoma sequence [42]. Dehghanizadeh et al. found that *BRAF* mutation correlates with a reproducible unique DNA methylation signature in sessile serrated polyps using exome sequencing [43]. The coexistence of *KRAS* and *BRAF* mutations may have profound clinical implications for disease progression and therapeutic responses [40, 44]. The activation of the MAPK pathway associates with the invasive behavior of several tumours and that hyperstimulation of several tyrosine kinases and MAPK has been found in solid. Prospective clinical trials including the inhibitor of the MAPK pathway, a potential clinical target, may be considered in the treatment of certain cancer [45]. So, there should be many different and complicated molecular mechanisms involved in epigenetic and genetic changes, which can affect cell behavior, cell environment, and contribute to the adenoma–carcinoma sequence. We found an epigenetic mechanism that the overexpression of DNMT3B may be involved in the formation of larger CAP. Genetic mechanism, such as *KRAS* and *BRAF* mutations and others, may be needed to clarify in bigger CAP in our further study.

## 5. Conclusion

*DNMT3B* overexpression is associated with the hypomethylation of *LINE-1* and the hypermethylation and silencing of *RASSF1A* expression in bigger CAPs. Our findings demonstrate that *DNMT3B* should play a critical role in the stepwise progression from normal to dysplastic epithelium. Considering that *DNMT3B* is a potential target of biomarker and chemoprevention, the results of this study may have considerable clinical implications.

## Figures and Tables

**Table 1:** Parameters of CAP.

Parameters	Values
Age	
Mean	57.16 ± 7.07
Range	39-69
Gender	
Male	13
Female	7
Male : female	1.86 : 1
Site	
Colon	9
Rectosigmoid colon	5
Rectal	6
Size	
Mean	13 mm
Range	10–19 mm
Histopathological types (%)	
Tubular	13 (65%)
Tubulovillous	6 (30%)
Villous	1 (5%)

**Table 2:** 

Name	Forward	Reverse
DNMT1	F: AACCTTCACCTAGCCCCAG	R: CTCATCCGATTTGGCTCTTTCA
DNMT3A	F: GACAAGAATGCCACCAAAGC	R: CCATCTCCGAACCACATGAC
DNMT3B	F: AGGGAAGACTCGATCCTCGTC	R: CGTCTCCGAACCACATGAC
P16	F: ATGGAGCCTTCGGCTGACT	R: GTAACTATTCGGTGCGTTGGG
hMLH1	F: TTCGTGGCAGGGGTTATTCG	R: GCCTCCCTCTTTAACAATCACTT
RASSF1A	F: AGGACGGTTCTTACACAGGCT	R: TGGGCAGGTAAAAGGAAGTGC
*β*-actin	F: CATGTACGTTGCTATCCAGGC	R: CTCCTTAATGTCACGCACGAT

**Table 3:** 

Name	Forward	Reverse
FI- hMLH1	F: GGTATTTTTGTTTTTATTGGTTGGAT	R: AATACCAATCAAATTTCTCAACTCCT
M-hMLH1	F: TAAAAACGAATTAATAGGAAGAGC	R: CTCTATAAATTACTAAATCTCTTCG
UM-hMLH1	F: TAAAAATGAATTAATAGGAAGAGT	R: CTCTATAAATTACTAAATCTCTTCA
FI-P16	F: GGAGAGGGGGAGAGTAGGT	R: CTACAAACCCTCTACCCACCT
M-P16	F: CGGGGAGTAGTATGGAGTCGGCGGC	R: GACCCCGAACCGCGACCGTAA
UM-P16	F: TGGGGAGTAGTATGGAGTTGGTGGT	R: CAACCCCAAACCACAACCATAA
FI-RASSF1A	F: GTTTAGTTTGGATTTTGGGGGAG	R: CCCRCAACTCAATAAACTCAAACT
M-RASSF1A	F: GGGTTCGTTTTGTGGTTTCGTTC	R: GATTAAACCCGTACTTCG
UM-RASSF1A	F: GGGGTTTGTTTTGTGGTTTTGTTT	R: AACATAACCCAATTAAACCCATACTTC

## Data Availability

The (western blot, real-time PCR, MS-PCR, ELISA, and COBRA) data used to support the findings of this study are included within the article.

## Supplementary Materials

Supplementary Materials S Figure 1: the absolute quantification of DNMTs expression levels in CAP and NCM tissues
