# Supplementary material for: DNMT3B Expression Might Contribute to Abnormal Methylation of RASSF1A in Lager Colorectal Adenomatous Polyps
Source: Gastroenterol Res Pract. 2020 Oct 1;2020:1798729. doi: 10.1155/2020/1798729 (PMC7547352; doi:10.1155/2020/1798729)
Supplement: Supplementary Materials — S Figure 1: the absolute quantification of DNMTs expression levels in CAP and NCM tissues [file 1798729.f1.docx]

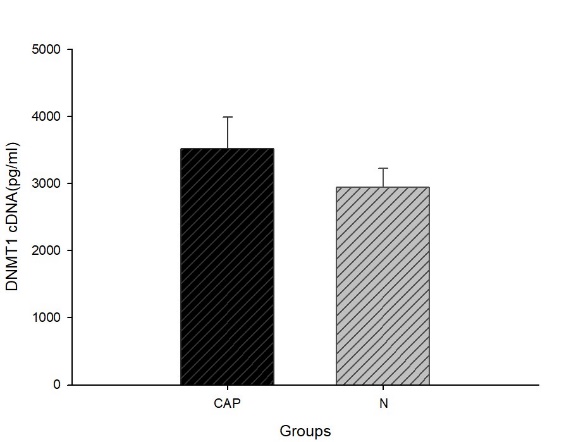

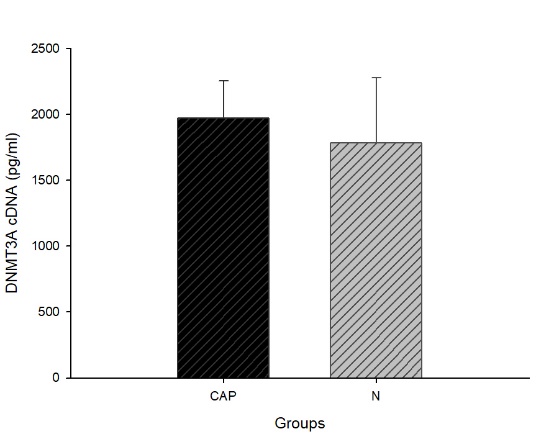

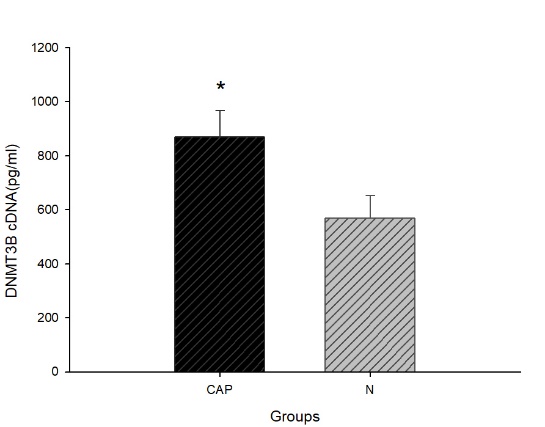


**B**

**C**

**A**

**S Figure 1. The absolute quantification of DNMTs expression levels in CAP and NCM tissues.**

A:DNMT1; B :DNMT3A; C: DNMT3B.

**S table 1. The intensity ratio of DNMTs/β-actin in CAP and NCM tissues**

| number | NDMT1 | | DNMT3A | | DNMT3B | |
| --- | --- | --- | --- | --- | --- | --- |
|  | CAP | N | CAP | N | CAP | N |
| 1 | 0.2569 | 0.1964 | 0.0312 | 0.0332 | 0.2685 | 0.111 |
| 2 | 0.2969 | 0.2729 | 0.0307 | 0.0123 | 0.3653 | 0.1739 |
| 3 | 0.2715 | 0.2443 | 0.0244 | 0.0279 | 0.1948 | 0.1899 |
| 4 | 0.2498 | 0.2764 | 0.0217 | 0.0297 | 0.1613 | 0.1620 |
| 5 | 0.2569 | 0.2529 | 0.0225 | 0.0299 | 0.3304 | 0.1777 |
| 6 | 0.2615 | 0.2262 | 0.0192 | 0.0247 | 0.2885 | 0.1088 |
| 7 | 0.2639 | 0.2964 | 0.0274 | 0.0208 | 0.2753 | 0.1227 |
| 8 | 0.2775 | 0.3029 | 0.0287 | 0.0190 | 0.2971 | 0.1544 |
| 9 | 0.3115 | 0.1943 | 0.0283 | 0.0217 | 0.2528 | 0.2626 |
| 10 | 0.3232 | 0.2887 | 0.0285 | 0.0254 | 0.1374 | 0.1591 |
| 11 | 0.2619 | 0.1964 | 0.0251 | 0.0251 | 0.2968 | 0.1795 |
| 12 | 0.3190 | 0.2897 | 0.0274 | 0.0267 | 0.1866 | 0.1885 |
| 13 | 0.2819 | 0.2411 | 0.0321 | 0.0271 | 0.2775 | 0.2754 |
| 14 | 0.2339 | 0.2532 | 0.0240 | 0.0250 | 0.2099 | 0.2189 |
| 15 | 0.2469 | 0.2595 | 0.0230 | 0.0249 | 0.3196 | 0.1322 |
| 16 | 0.2685 | 0.1987 | 0.0239 | 0.0287 | 0.3053 | 0.1577 |
| 17 | 0.2126 | 0.2906 | 0.0291 | 0.0290 | 0.2365 | 0.2355 |
| 18 | 0.2477 | 0.3009 | 0.0245 | 0.0275 | 0.3215 | 0.1105 |
| 19 | 0.3001 | 0.1950 | 0.0317 | 0.0207 | 0.2885 | 0.1542 |
| 20 | 0.2982 | 0.3159 | 0.0282 | 0.0251 | 0.3215 | 0.1470 |
